# Congenital clitoromegaly in an adult

**DOI:** 10.11604/pamj.2019.34.141.18149

**Published:** 2019-11-12

**Authors:** Mohammed Alae Touzani, Imad Ziouziou

**Affiliations:** 1 Department of Urology « B », Avicenne Hospital, Rabat, Morocco; 2 Department of Urology, Hassan II Hospital, Ibn Zohr University, Agadir, Morocco

**Keywords:** Clitoris, clitoromegaly, 21 hydroxylase, pseudo-hermaphroditism

## Image in medicine

Clitoromegaly is an abnormal enlargement of the clitoris. In adult, dimensional criteria are, according to Brodie, a minimum length hood and width of 27.4mm and 8mm. We here report the case of a 26 year female patient, with no previous personal history, who consults for primary amenorrhea with androgenization signs (hirsutism, deep voice…). Clinical examination showed a clitoromegaly and urogenital sinus persistency. The patient first underwent a karyotype that shows a female karyotype with no abnormalities (46,XX). Further explorations showed a 21-hydroxylase deficiency, with a congenital bilateral adrenal hyperplasia. Congenital adrenal hyperplasia (CAH) is defined by an increased size and metabolism of the adrenal glands due to enzymatic disorders, such as 21-hydroxylase deficiency. In women, this can lead to virilization of the external genitalia in 11% of patients, and a late diagnosis, such in this case, may result in the late discovery of androgenization signs, such as clitoromegaly. The treatment is clitoroplasty, which consist to reduce the clitoris size and preserve its sensitivity, with a vaginoplasty if there is an association with urogenital sinus persistency, if the patient is asking for it.

**Figure 1:**
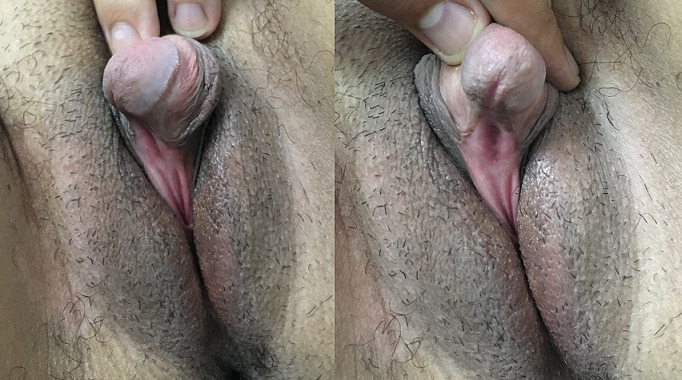
Clitoromegaly with a “male glans” aspect

